# Aptamer Detection of *Mycobaterium tuberculosis* Mannose-Capped Lipoarabinomannan in Lesion Tissues for Tuberculosis Diagnosis

**DOI:** 10.3389/fcimb.2021.634915

**Published:** 2021-03-15

**Authors:** Yuanyuan Zhou, Huan Xiong, Rong Chen, Lixia Wan, Ying Kong, Jianwei Rao, Yan Xie, Chaolin Huang, Xiao-Lian Zhang

**Affiliations:** ^1^ State Key Laboratory of Virology and Hubei Province Key Laboratory of Allergy and Immunology, Department of Immunology, Wuhan University School of Basic Medical Sciences, Wuhan, China; ^2^ Department of Allergy, Zhongnan Hospital, Wuhan University, Wuhan, China; ^3^ Medical Research Institute, Frontier Science Center for Immunology and Metabolism, Wuhan University, Wuhan, China; ^4^ Department of Pathology, Medical Department, Jin Yin-Tan Hospital, Wuhan, China; ^5^ Wuhan Research Center for Communicable Disease Diagnosis and Treatment, Chinese Academy of Medical Sciences, Wuhan, China

**Keywords:** aptamer, ManLAM, tuberculosis, diagnosis, lesion tissue

## Abstract

Tuberculosis (TB) is the leading infectious cause of mortality worldwide. However, the diagnosis of TB, especially extrapulmonary TB (EPTB) diagnosis from lesion tissues, remains a challenge. Nucleic acid aptamers are analogous to antibodies and have advantages of easier modification, high specificity, and affinity. Mannose-capped lipoarabinomannan (ManLAM) is a unique surface lipoglycan component or constantly released from *mycobacterium tuberculosis* (*M.tb)* cell wall, which makes it a perfect candidate biomarker for TB diagnosis. Our present study aims to establish *M.tb* ManLAM aptamer-based immunohistochemistry (IHC) method for TB diagnosis. We performed TB diagnosis using 263 formalin-fixed paraffin-embedded tissue samples including 213 TB samples (pulmonary TB (PTB) and EPTB), and 8 samples from latent TB infection (LTBI) high risk subjects, and 42 samples from other non-TB patients with ManLAM aptamer-based IHC and routine laboratory TB diagnostic methods parallelly. The sensitivity and specificity of the ManLAM aptamer-based IHC were 86.38% and 92.86%, with much higher sensitivity than those of mycobacterial culture (9.66%) and acid-fast staining (AFS) (43.01%) and comparability to Interferon-gamma Release Assay (IGRA) (84.38%) and GeneXpert (79.31%). High agreement between ManLAM based-IHC and IGRA or GeneXpert for TB diagnosis were observed. Furthermore, ManLAM aptamer-based IHC combination with other routine TB laboratory diagnostic methods significantly increased the sensitivity up to 88.64%–97.92%. As our knowledge, this is the first report about aptamer-based IHC for disease diagnosis. Thus, ManLAM aptamer-based IHC has potentials for TB diagnosis, including PTB, and EPTB, and assists the diagnosis of LTBI with high effectiveness, feasibility, and easy production.

## Introduction

Tuberculosis (TB), caused by *Mycobacterium tuberculosis* (*M.tb* or MTB), is one of the top 10 causes of death and the leading killer from a single infectious agent worldwide. One quarter of people in the world have been infected with *M.tb*. The World Health Organization (WHO) has reported that TB causes an approximately 1.5 (1.4–1.6) million deaths in 2018 (World Health Organization, 2019a). *M.tb* usually infects the lungs and causes pulmonary TB (PTB), but it can also infect lymph nodes, bone, and other tissues causing extrapulmonary TB (EPTB). *M.tb* can also exist in host body without causing any clinical symptoms (latent TB infection, LTBI), thus could form new infection sources when latent *M.tb* bacteria were reactivated. The nontuberculous mycobacteria (NTM) patients were subjects infected with mycobacteria not causing tuberculosis and leprosy. Early diagnosis of TB, EPTB, and LTBI is necessary for effective treatment of TB.

Currently, diagnosis of TB, especially EPTB remains a critical challenge. Although many approaches have been developed for laboratory TB diagnosis so far, including *M.tb* culture, the acid-fast bacilli staining (AFS), the antibody detection, interferon-gamma release assay (IGRA) and the GeneXpert MTB/RIF, a definite diagnosis is based on the presence of acid-fast bacilli and/or isolation of *M. tb* in culture from biopsy specimens or fine-needle aspirates. Usually for clinicians or surgical pathologist, it is very difficult for them to determine whether the suspected lesions including tumor lesions are caused by *M.tb* infection, LTBI, NTM, or other pathological changes. Staining for acid-fast bacilli (AFS) has low sensitivity and could not differentiate mycobacterial species either. The detection limit for AFS is > 10^4^ bacilli per slide, or 10^4^ bacilli per ml of specimen (Ulrichs et al., 2005; Datta and Evans, 2020). A diagnosis is therefore usually made on the basis of the classical histological changes of chronic granulomatous inflammation suggestive of tuberculosis. Most cases of EPTB are paucibacillary. Mycobacterial culture takes several weeks and its sensitivity is also low in paucibacillary conditions (Mishra et al., 2005; Wallis et al., 2010; Calligaro et al., 2017). Thus, there are samples for EPTB that are both acid-fast bacilli- and culture-negative. These histological features, such as granuloma and chronic inflammatory cell infiltration, can be found in various conditions and diseases other than tuberculosis. The histological features can be atypical in immuno-compromised tuberculosis patients, leading to considerable difficulty and delay in diagnosis (Jain et al., 2017; Mehta et al., 2019). It is not possible to differentiate between mycobacterial species based on histology alone. The management (drug doses and combinations) is different for diseases caused by *M. tb* and non-tuberculous mycobacterial infection (Lopez-Varela et al., 2015; Wu and Holland, 2015). An incorrect diagnosis of tuberculosis leads to increased morbidity and mortality due to suboptimal or wrong treatment and has significant economic implications. Thus, it is urgent to develop new methods to improve the diagnostic tests for PTB and EPTB, and to determine the type of mycobacterium involved, for better clinical management.

Nucleic acid aptamers are short, single-stranded oligonucleotides that fold into particular structures to bind to target molecules such as small chemicals, sugars, lipids, proteins, or cells (Ellington and Szostak, 1990; Levay et al., 2015). High-affinity aptamers for specific target molecules can be isolated from a random nucleic acid library *in vitro* using the systematic evolution of ligands by exponential enrichment (SELEX) (Ellington and Szostak, 1990; Levay et al., 2015). Aptamers are analogous to antibodies, but possess several key advantages, such as easier production and modification, higher stability, specificity, and affinity, making aptamers promising tools in analytical and diagnostic applications (White et al., 2000). A large number of researches have utilized aptamers for infection diagnosis, either by aptamers detecting pathogenic toxins or proteins, or detecting pathogenic whole cells (Lavu et al., 2016; Park, 2018).

Mannose-capped lipoarabinomannan (ManLAM) is a unique surface lipoglycan component from *M.tb* cell wall. ManLAM can be constantly released from metabolically active or degrading mycobacteria into circulatory system, potentially in large amounts, which makes it a perfect candidate biomarker for TB diagnostic tests (Lawn, 2012; Tang et al., 2016; Paris et al., 2017). Previously, we have generated a single strand DNA aptamer (T9) “antibody” specific for *M.tb* glycolipid antigen ManLAM from Beijing genotype virulent strain by the SELEX (Tang et al., 2016). The selected single aptamer T9 demonstrated the highest specificity and binding affinity to ManLAM with serum or sputum samples (Tang et al., 2016).

In this study, we used ManLAM aptamer-based immunohistochemistry (IHC) to detect ManLAM from the lesion tissue specimens from TB and EPTB patients and non-TB controls. The aim of this study is to establish an aptamer-based IHC method for detecting the tuberculosis antigenic biomarker ManLAM in lesion tissues. We hope our established method could resolve the diagnosis difficulty faced by clinicians or pathologist for determining whether the suspected lesions including tumor lesions are caused by *M.tb* infection, LTBI, NTM, or other pathological changes. Our research here demonstrated that ManLAM aptamer-based IHC analysis can be used for TB diagnosis including PTB, EPTB and TB with immunodeficiency, and assist the diagnosis of LITB with high effectiveness, feasibility and easy production. Its combination with other routine laboratory TB diagnostic methods can remarkably improve the sensitivity of TB diagnosis.

## Materials and Methods

### Ethics Statement and Participant Inclusion Criteria

The research related to human samples used has been complied with all the relevant national regulations, institutional human experimental guidelines of Wuhan University School of Medicine and Jin Yin-Tan Hospital, Wuhan, and in accordance the tenets of the Helsinki Declaration. The study was approved by the ethics committee of Wuhan University School of Medicine and Jin Yin-Tan Hospital, Wuhan (No. AF/SC-08/02.0, KY-TB-2019-01.01) and conducted following the human experimental guidelines of Wuhan University School of Medicine and Jin Yin-Tan Hospital, Wuhan. All animal experimental protocols were performed in compliance with the Chinese National Laboratory Animal-Guideline for Ethical Review of Animal Welfare and approved by the Institutional Animal Care and Use Committee (IACUC) of Wuhan University (No. 18021B). The test subjects were enrolled in 2018 from Jin Yin-Tan Hospital, Wuhan, Hubei province, China. The diagnosis methods of TB were performed according to a composite reference standard (CRS), which combined the WHO guidelines (World Health Organization, 2019b) and WS 288-2017 by the National Health Commission of the People’s Republic of China (2019). All of the subjected were unrelated Chinese of the Han nationality. Formalin-fixed lesion tissues from patients were paraffin-embedded, and cut into 5~7 μm sections.

### Criteria for Subject Inclusion

Briefly, the criteria are as follows: (1) microbiologically confirmed PTB: patients with positive MTB culture, or a pulmonary case with one or more positive initial sputum smears and chest imaging; (2) molecular biologically confirmed PTB: patients with positive MTB nucleic acid test and chest imaging; (3) histopathological examination confirmed PTB: patients with positive histopathological examination; (4) EPTB: patients with definite TB involving organs other than the lungs, with MTB isolated from a non-pulmonary source or histological or strong clinical evidence consistent with active EPTB, as well as improvement observed in anti-TB specific therapy. Thus, CRS was a combination of many pertinent aspects (Li et al., 2020). Non-TB samples have been identified by a lack of symptoms of TB, no TB presentation on the X-ray examination, an epidemiologically low risk of TB infection (no contact with TB), and no TB associated histochemistry presentations. The nontuberculous mycobacteria (NTM) patients were subjects infected with mycobacteria not causing tuberculosis and leprosy. NTM was identified by para-nitrobenzoic acid (PNB) test and Mycobacteria Species Identification Genetic Detection Kit (Yaneng Bio, China). The latent TB infection (LTBI) suspects were defined as subjects with *M.tb* exposure risk and showed positive results in IGRA assay, but showed no TB clinical symptoms. Healthy donors (HDs) did not include TB suspects later ruled out for TB.

### M.tb Culture

The sputum samples (5 ml) were transferred into a 50 ml centrifuge test tube, and mixed with an equal amount of 2% N-Acetyl-L-Cysteine sodium hydroxide. Then 50 ml sterile phosphate buffered saline (PBS) (pH 6.8) were added. Samples were centrifuged at 3000 × g for 15 min and the supernatant was discarded. At last, 0.5 ml of the culture specimens were placed in BACTEC™ MGIT 320 (BD) and incubated.

### Acid-Fast Bacilli Staining (AFS)

The AFS kits using Ziehl-Neelsen (ZN) staining were purchased from Baso Diagnostics Inc (Zhuhai, China). The experiment was carried out according to instructions of the kit. Briefly, 5~7 μm sectioned specimens, were incubated at 60°C for 1 h, then deparaffinized and rehydrated with consecutive dilutions of alcohol (absolute ethanol, 95% ethanol, 85% ethanol, and 75% ethanol). Slides were stained with carbolfuchsin for 10 min at room temperature, decolorized with acidified alcohol, and then counterstained with methylene blue for 2 min at room temperature (Che et al., 2016).

### Interferon-Gamma Release Assay (IGRA)

The IGRA assay was performed according to the manufacture’s clinical protocol of *Mycobacterium Tuberculosis* Specific Cell Mediated Immune Response Detection Kit (HG-IGRA, Wuhan Hygeianey Bioscience Co., Ltd.).

### GeneXpert M. TB/RIF Assay

Xpert MTB/RIF assay was performed on the GeneXpert Dx instrument system according to the manufacturer’s recommendations (Cepheid, Sunnyvale, CA, USA). Briefly, samples were treated with sample reagent (SR) containing NaOH and isopropanol. The SRs were added at a 2 to 1 ratio of the sputum samples, and the samples were homogenized and incubated for 15 min at room temperature. The treated samples were transferred into the cartridge of the GeneXpert instrument (Scott et al., 2011; Geleta et al., 2015). The Xpert software was used to interpret the results, and semiquantitative results were provided based on the cycle threshold (C_T_) defined by the manufacturer as follows: high (C_T_ ≤ 16), medium (16 < C_T_ ≤ 22), low (22 < C_T_ ≤ 28), and very low (C_T_ > 28).

### IHC Staining

IHC staining utilizing biotin-labeled ssDNA T9 aptamer against ManLAM (Tang et al., 2016) was performed by researchers in advance unaware of clinical information and reference test results. Random sectioned specimens (5~7 μm) were incubated at 60°C for 1 h, then deparaffinized and rehydrated with consecutive dilutions of alcohol (absolute ethanol, 95% ethanol, 85% ethanol and 75% ethanol). After antigen retrieval, all the samples were incubated with hydrogen peroxide at room temperature for 10 min to inhibit the endogenous peroxidase activity. Next, samples were blocked with 100 μl 5% BSA at room temperature for 30 min, and 100 μl 300 nM biotin-labeled ssDNA aptamer or rabbit anti-Rv2645 (1:300) (Luo et al., 2015), anti-Rv1579 (1:500) (Qu et al., 2020) or commercial anti-Ag85B polyclonal antibody (Abcam, ab43019, 1:100) was added and incubated at 37°C for 1 h. Horseradish peroxidase (HRP)-conjugated streptavidin (YEASEN, 35105ES60, 1:500) or HRP-anti-rabbit IgG (Proteintech, SA00001-2, 1:500) was added and incubated for 30 min at 37°C. 3,3′-diaminobenzidine tetrahydrochloride (DAB) Chromogen Solution was added for 1 min, then sections were counterstained with hematoxylin, dehydrated, and then sealed with neutral resin glue. Images were analyzed by using a FISH multi-function scanner (Aperio VERSA 8, Leica) or observed under normal optical microscope. The IHC scores were calculated by ImageJ software (Schneider et al., 2012) with Bio-Formats (Linkert et al., 2010) and IHC Profiler plugins (Varghese et al., 2014) which accounted for the background (IHC Score = [Percentage of High Positive Pixels] × 4 + [Percentage of Positive Pixels] × 3 + [Percentage of Low Positive Pixels] × 2 – [Percentage of Negative Pixels]) (Varghese et al., 2014). The calculated IHC scores were introduced to assess the degree of positive staining, and the method had taken background signal into consideration, thus, to a certain extent, eliminated the interference of background.

### Immunofluorescence (IF) Staining

IF staining was performed by utilizing AF488-labeled ssDNA T9 aptamer against ManLAM (Tang et al., 2016). Sectioned specimens (5~7 μm) were incubated at 60°C for 1 h, then deparaffinized and rehydrated with consecutive dilutions of alcohol (absolute ethanol, 95% ethanol, 85% ethanol and 75% ethanol). After antigen retrieval, samples were blocked with 100 μl 5% BSA at room temperature for 30 min, then 100 μl 300 nM AF488-labeled ssDNA aptamers or AF488 labeled anti rabbit-IgG against rabbit anti-Rv2645 (1:300) and anti-Rv1579 polyclonal antibodies (1:500) were used to detect the *M.tb* antigens in the lung sections from *M.tb* infected mouse were added and incubated at 37°C for 1 h. Lastly, sections were sealed with Anti-fade Fluorescence Mounting Medium. Images were analyzed by using a FISH multi-function scanner (Aperio VERSA 8, Leica) or using a normal fluorescence microscope.

### M.tb Infection Mouse Model

Each mouse was infected with *M.tb* standard virulent strain H37Rv (1 × 10^6^ CFU bacteria/mouse) through intranasal infection on Day 0. After infection for 28 days, each mouse was sacrificed and lungs from each infected mouse were obtained for lung tissue sections analysis.

### Statistical Analysis

The statistical analysis was performed using GraphPad Prism V.5.00 software (GraphPad Software, San Diego CA, USA). Statistical difference between two groups were assess by Mann-Whitney U test. Statistical proportion test (using R prop.test) was used to statistically evaluate the detection rates obtained by different diagnostic methods (Newcombe, 1998). McNemar Chi-test with Yate’s correction for continuity was used for test of agreement between two different methods. A *P* value less than 0.05 was considered statistically significant. The receiver-operator-characteristics (ROC) of the scores for IHC assay were plotted and the area under the curves (AUC) was calculated. The optimal cutoff values were chosen as when Youden’s index (YI = sensitivity+specificity-1) was maximum (Obuchowski and Bullen, 2018). Sensitivity was calculated as the proportion of test-positive TB patients in all TB patients. Specificity was calculated as the proportion of test-negative non-TB patients in all non-TB patients. AUC values between 0.8 and 0.9 were deemed “accurate”; 0.7-0.8, “moderately accurate”; 0.5-0.7, “uninformative” (Swets, 1988).

## Results

### Aptamer-Based Immunohistochemistry TB Diagnosis for ManLAM With Much Higher Sensitivity and Specificity Than Mycobacterial Culture and AFS

In this study, we collected lesion tissues from 263 subjects with a confirmed diagnosis of TB (n = 213, 121 male/92 female, age 31.0 (0.2–86.0) years, including 38 PTB and 175 EPTB), LTBI (n = 8, four male/four female, age 59.5 (44.0–71.0) years) or non-TB (n = 42, 30 male/12 female, age 48.5 (1.0–72.0) years) (**Supplementary Table 1** and **Supplementary Figure 1**).

The formalin-fixed paraffin-embedded tissue sections of the 263 samples were detected by the ManLAM based-IHC assay as described in *Materials and Methods*. Briefly, each sectioned specimen (263 specimens) was incubated biotin-labeled ManLAM aptamer, subsequently with addition of HRP-streptavidin, and developed with DAB Chromogen Solution, then counterstained with hematoxylin. Finally, images of each 263 specimens were analyzed by using a FISH multi-function scanner with optical microscope.

**Figure 1:**
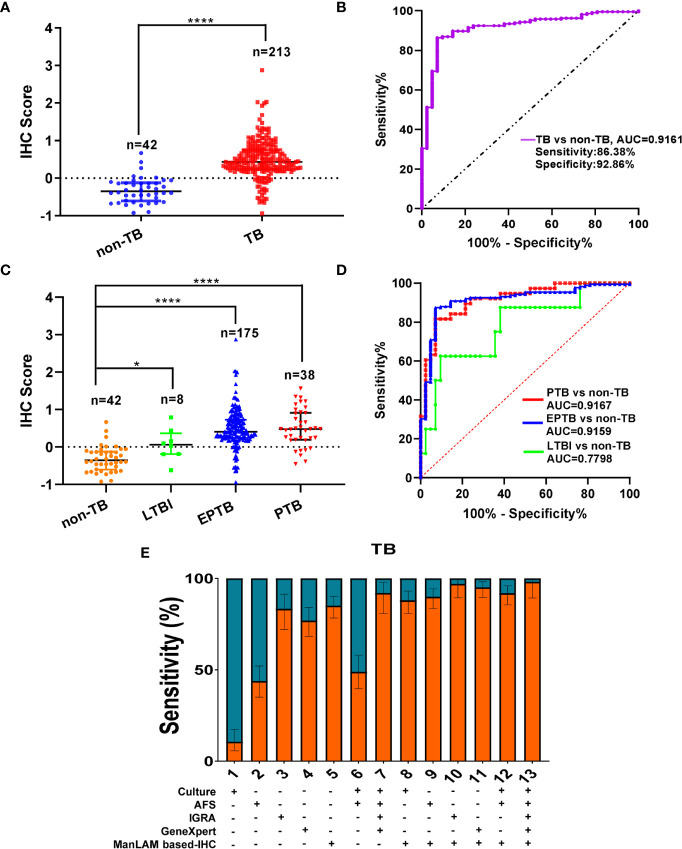
Much higher immunohistochemistry (IHC) scores in tuberculosis (TB) patients than those in non-TB patients. **(A)** Comparison of IHC scores between non-TB and TB patients, cutoff value: 0.0580 (dotted line). Data are presented as median ± IQR (inter-quantile range). **(B)** ROC curve for IHC score for TB patients vs. non-TB patients. **(C)** Comparison of IHC scores between non-TB and LTBI/PTB/EPTB patients. **(D)** ROC curve for IHC score for latent TB infection (LTBI) patients vs. non-TB patients, PTB patients vs. non-TB patients, and EPTB patients vs. non-TB patients. **(E)** The sensitivities of ManLAM based-IHC, Culture, AFS, IGRA, GeneXpert, or their combination for TB patients. The sensitivities were statistically evaluated with statistical proportion test (R prop.test). *P < 0.05, ****P < 0.0001, (Mann-Whitney U test, vs. non- TB) in **(A, C)**.

We used ImageJ/IHC profiler software to analyze all the results by the aptamer based-IHC. We found that TB patients (n = 213) showed significantly higher IHC scores (median: 0.4319, inter-quantile range-IQR 0.2355~0.7560) compared with non-TB patients (n = 42, median: -0.3505, IQR: -0.6054 ~ -0.1233) (**** *P* < 0.0001, **Figure 1A**, cutoff value: 0.0580). Receiver operating characteristic (ROC) curves were used to evaluate the performance of IHC score for TB diagnosis. The area under the ROC curve (AUC) of the IHC for TB vs non-TB was 0.9161 (95% confidence interval-CI: 0.8712 to 0.9609), with a sensitivity of 86.38% (95%CI = 80.87%–90.55%) (**Figure 1B**), while the sensitivity of AFS was calculated as 43.01% (95%CI = 35.98%–50.32%) (**Table 1**), whereas the sensitivity of Culture was calculated as 9.66% (95%CI = 5.90%–15.26%) (**Table 1**). We further measured the sensitivity of IGRA as 84.38% (95%CI: 75.22%–90.70%), while the sensitivity of GeneXpert was calculated as 79.31% (95%CI: 72.53%–85.07%) (**Table 1**). These data strongly suggest that the sensitivity and detection accuracy of the ManLAM aptamer-based IHC (86.38% and 87.45%) were much higher than those of mycobacterial culture (9.66%, 16.32%) and AFS (43.01%, 47.37%), and similar to IGRA (84.38%, 85.98%) and GeneXpert (79.31%, 79.78%) (**Table 1**).

**Table 1:** Diagnostic performance of ManLAM aptamer based-immunohistochemistry (IHC) and other methods for tuberculosis (TB) diagnosis.

TB vs non-TB	Accuracy (95%CI)	Sensitivity (95%CI)
IHC	87.45% (82.75%–91.26%)	86.38% (80.87%–90.55%)
Culture	16.32% (11.36%–22.35%)	9.66% (5.90%–15.26%)
AFS	47.37% (40.44%–54.37%)	43.01% (35.98%–50.32%)
IRGA	85.98% (77.93%–91.94%)	84.38% (75.22%–90.70%)
GeneXpert	79.78% (73.23%–85.35%)	79.31% (72.53%–85.07%)

95%CI, 95% confidence interval; the 95% CIs shown in parentheses were generated by bootstrapping. NA, not available. Cutoff values were determined using Youden’s Index, i.e., the maximum of (sensitivity + specificity - 1). Accuracy (Correct classification rate) = (No. of Test positive TB patients + No. of Test negative non-TB)/(No. of TB patients + No. of non-TB); Sensitivity = (No. of Test positive TB patients)/(No. Of TB patients); Specificity = (No. of Test negative non-TB)/(No. of non-TB).

Further, the AUC for LTBI (8 samples) vs non-TB (42 samples including 2 NTM) was 0.7798 (95% CI: 0.5950 - 0.9645) with a sensitivity of 62.50% (95% CI: 30.57% to 86.32%) and a specificity of 90.48% (95% CI: 77.93% to 96.23%) (**Figure 1C**), while the AUC for PTB vs non-TB was 0.9167 (95% CI: 0.8556 to 0.9777) with a sensitivity of 81.58% (95% CI: 66.58% to 90.78%) and a specificity of 92.86% (95% CI: 80.99% to 97.54%). Moreover, the AUC for EPTB vs non-TB was 0.9159 (95% CI: 0.8693 to 0.9626) with a sensitivity of 87.43% (95% CI: 81.70% to 91.55%) and a specificity of 92.86% (95% CI: 80.99% to 97.54%) (**Figures 1C, D**). Our present results demonstrated that similar AUC value and sensitivity were observed between EPTB and PTB *vs* non-TB (**Figure 1D**). Above data suggest that aptamer-based IHC TB diagnosis could be used for TB diagnosis including PTB, EPTB, and LTBI, and has high sensitivity and specificity for PTB and EPTB diagnosis.

We further combined ManLAM based-IHC with other routine laboratory TB diagnosis methods to assess whether this combination could further enhance the diagnostic performance (**Supplementary Table 2**, **Figure 1E**). The results showed that the sensitivities were increased from 9.66% for culture to 88.64% for culture plus IHC, from 43.01% for AFS to 91.19% for AFS plus IHC, from 84.38% for IGRA to 97.92% for IGRA plus IHC, and 79.31% for GeneXpert to 95.98% for GeneXpert plus IHC (**Supplementary Table 2**, **Figure 1E**). Whilst the sensitivities were improved from 4.10% for AFS plus Culture to 90.77% when IHC combined with AFS plus Culture, and from 83.90% for AFS, Culture, IGRA plus GeneXpert to 97.07% when IHC combined with AFS, Culture, IGRA plus GeneXpert MTB/RIF **(** **Supplementary Table 2**, **Figure 1E**). We performed statistical evaluation of the sensitivities with statistical proportion test using R software (prop.test function) (**Supplementary Table 2**) according to previous publication (Newcombe, 1998). The sensitivities after combination of ManLAM based-IHC with culture, AFS, IGRA and GeneXpert MTB/RIF were significantly increased with remarkable differences compared to those routine TB diagnostic approaches (*P* < 0.0001 as shown in **Supplementary Table 2**, except for IHC combination with IGRA group, *P* < 0.05).

### Detection of Clinical TB and Non-TB Samples by IHC With ManLAM Aptamer

**Figure 2:**
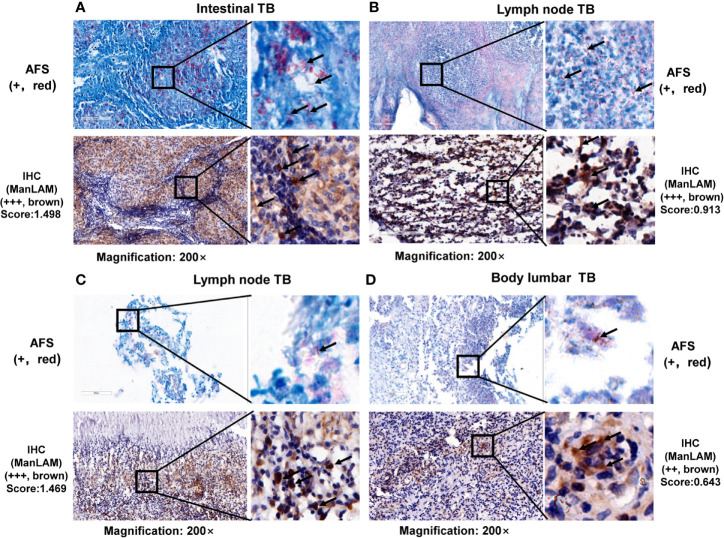
Representative images and comparison between acid-fast staining and ManLAM-based immunohistochemistry (IHC) on lesion tissues. Representative images of **(A)** lesion sections of intestinal tuberculosis (TB) patient, **(B, C)** tissues of lymph node TB patients, and **(D)** lesion sections of body lumbar TB. TB, tuberculosis. Black arrows indicate positive signals (AFS by red staining, or aptamer-based IHC by brown granular staining). Samples with a score less than the cutoff value (0.0580) were designated as “-”, (≥0.0580 and <0.5010) as “+”, (≥0.5010 and <0.8780) as “++”, >0.8780 as “+++”. The numbers (0.5010 and 0.8780) were determined by calculating the 50% and 75% percentile of the Scores.

**Figure 3:**
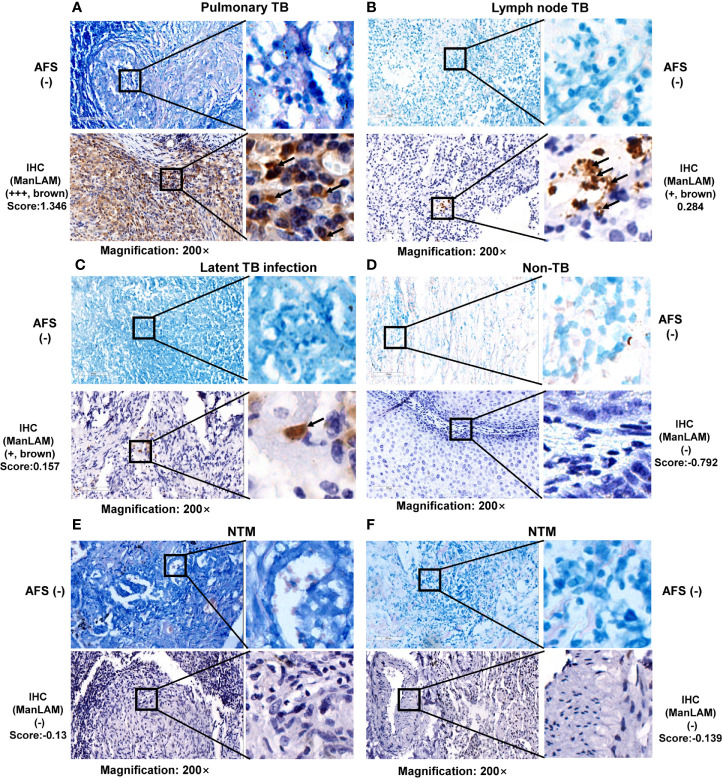
Representative images and comparison between acid-fast staining (AFS) and ManLAM aptamer-based IHC in lesion tissues with negative AFS. Representative images of **(A)** tissues of pulmonary tuberculosis (TB) patient, **(B)** lesion sections of lymph node TB patient, **(C)** lesion sections of LTBI patient, **(D)** lesion sections of non-TB patient, **(E, F)** lesion sections of two NTM patients. TB, tuberculosis. NTM, nontuberculous mycobacteria. Arrows indicate positive signals (aptamer-based immunohistochemistry (IHC) by brown granular staining; AFS by red staining). Samples with a score less than the cutoff value (0.0580) were designated as “-”, (≥0.0580 and <0.5010) as “+”, (≥0.5010 and <0.8780) as “++”, >0.8780 as “+++”. The numbers (0.5010 and 0.8780) were determined by calculating the 50% and 75% percentile of the Score.

Representative results are shown in **Figures 2**, **3**. By scanning different tissue slices or analyzed under optical microscope, we observed that ManLAM antigen could be detected in the lesion tissues with aptamer-based IHC in PTB and EPTB (**Figures 2** and **3**). ManLAM-based IHC staining from intestinal TB (**Figure 2A**), two lymph node TB (LNTB) specimens (**Figures 2B, C**), and a body lumbar TB (**Figure 2D**) had positive staining signals indicated by brownish yellow or brown color (lower panels shown in **Figures 2A–D**) when AFS (upper panels in **Figures 2A–D**) methods, parallelly, were also highly positive stained. We could even observe strong ManLAM signals (+++, brownish yellow) with high scores in the granuloma tissues of one intestinal TB patient and one lymph node TB patient (**Figures 2A, B**).

Furthermore, the IHC results were positive for one PTB patient (**Figure 3A**), one LNTB patient (**Figure 3B**) and one LTBI patient (**Figure 3C**) even when AFS results were negative for the same samples as shown in **Figures 3A–C**, suggesting that ManLAM-based IHC is more sensitive than the AFS method.

Furthermore, ManLAM signal by IHC was negative in the lesion section of one non-TB patient (a patient with perianal abscess of condyloma acuminatum) (**Figure 3D**), and no ManLAM signals were observed either in the lesion sections of two NTM patients **(** **Figures 3E, F**), while AFS staining were also negative (**Figures 3E, F**). Above results suggest that ManLAM-based IHC method is specific for TB diagnosis.

### High Agreement Between ManLAM Based-IHC and IGRA or GeneXpert MTB/RIF for TB Diagnosis

Then, we analyzed 96 patients with both ManLAM based-IHC and IGRA TB assays. Out of 96 TB patients, 81 had positive results by IGRA, whereas 85 had positive results by ManLAM aptamer based IHC. However, this difference was found to be no significance (corrected χ^2^ = 0.41, *P* = 0.5224) (**Table 2**), indicating that our IHC method is consistent with those of IGRA. In **Table 2**, 13 patients were positive in the ManLAM based-IHC but showed negative results in the IGRA. Further inspection of clinical information showed that eight of these 13 patients showed immunocompromised conditions: less than 1 year of age, suggesting that ManLAM aptamer-based IHC could be used for detecting TB in infant patients or immunodeficient patients, which is not suitable for the IGRA (**Supplementary Table 3**).

**Table 2:** High agreement between ManLAM aptamer based-immunohistochemistry (IHC) and IGRA/GeneXpert for tuberculosis (TB) diagnosis.

		ManLAM aptamer based-IHC		Total (n)			ManLAM aptamer based-IHC		Total (n)
		+	-				+	-	
**IGRA**	**+**	72	9^c^	81	**GeneXpert**	**+**	121	17^c^	138
	**-**	13^a^	2	15		**-**	29^b^	7	36
**Total (n)**		85	11	96	**Total (n)**		150	24	174

No significant differences of the positive rates were found between our method and IGRA or GeneXpert method (McNemar Chi-test with Yate’s correction for continuity, IGRA: χ2 = 0.41, P = 0.5224>0.05; GeneXpert: χ2 = 3.13, P = 0.0768>0.05).

^a^Eight of these 13 patients showed immunocompromised conditions: less than 1 year of age.

^b^For these 29 patients, 23 of these 29 patients were negative for AFS and 28 of these 29 patients was negative for mycobacterial culture.

^c^For these subjects, six out of these nine subjects who were negative in the ManLAM based-IHC but showed positive results in the IGRA, and 10 out of these 17 subjects who were negative in the ManLAM based-IHC but showed positive results in the GeneXpert had paucibacillary load and insufficient fine needle sampling of EPTB lesions.

We further analyzed 174 patient samples with ManLAM based-IHC and GeneXpert MTB/RIF. The sensitivity for TB was 79.31% (138/174) and 86.21% (150/174) by the GeneXpert MTB/RIF and ManLAM based-IHC method, respectively. However, this difference was found to be no significance (corrected χ^2^ = 3.13, *P* = 0.0768) (**Table 2**), indicating that the results obtained using our method has similar sensitivity with those obtained by GeneXpert MTB/RIF. Above results suggest that high agreement was observed between ManLAM based-IHC and IGRA or GeneXpert MTB/RIF for TB diagnosis. In **Table 2**, 29 patients were positive in the ManLAM aptamer based IHC, but showed negative results in the GeneXpert. Further inspection showed that 23 of these 29 patients were negative for AFS and only one of these 29 patients (**Supplementary Table 4**) was positive for bacterial culture. Thus, the negative GeneXpert results could be due to the sample processing quality, and patients with paucibacillary disease often caused false negative results in GeneXpert (Luabeya et al., 2019).

In **Table 2**, nine patients were negative in the ManLAM based-IHC but showed positive results in the IGRA, and 17 patients were negative in the ManLAM based-IHC but showed positive results in the GeneXpert. Further inspection of clinical information showed that eight of these nine patients (**Supplementary Table 5**) and 13 of these 17 patients (**Supplementary Table 6**) were EPTB. The possible reason for these negative ManLAM based-IHC results may be that the paucibacillary load of EPTB lesions and insufficient fine needle sampling of EPTB lesions led to insufficient or no ManLAM antigens in the tested lesion samples.

### Comparison of Aptamer- and Antibodies-Based IHC/Immunofluorescence for TB Diagnosis

**Figure 4:**
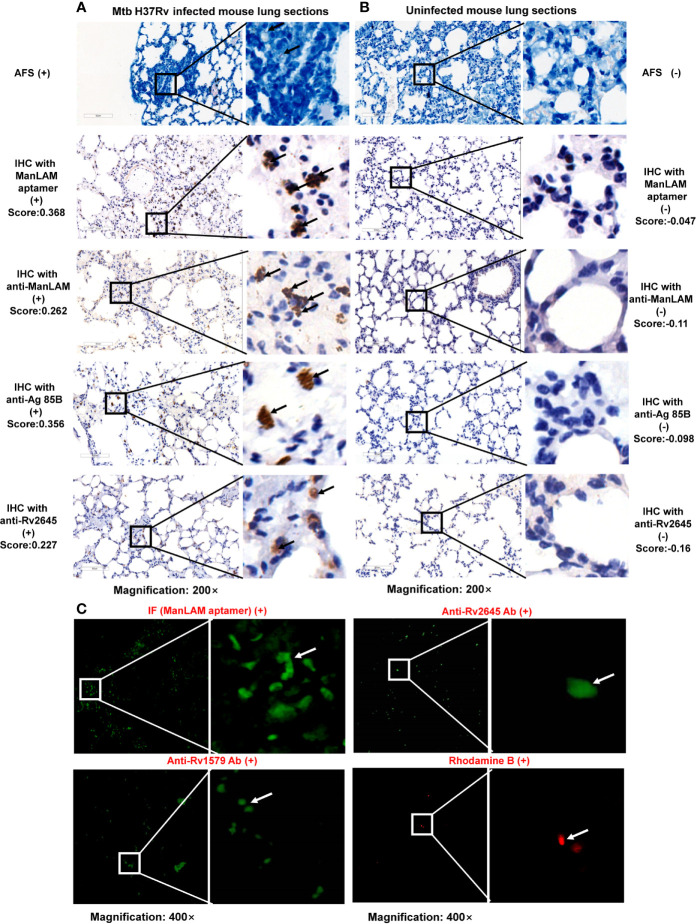
Comparison of aptamer- and antibodies-based immunohistochemistry (IHC)/immunofluorescence for tuberculosis (TB) diagnosis in TB mouse model. Images of *M.tb* H37Rv infected mouse lung sections **(A)** and uninfected mouse lung sections **(B)** detected by acid-fast staining (AFS), aptamer-based IHC, anti-ManLAM, anti-Ag85B, or anti-Rv2645 pAbs. Arrows indicate positive signals (aptamer-based IHC by brown granular staining; AFS by red staining). **(C)**. Images of infected mouse lung sections detected by AF488 labeled ManLAM aptamer-based immunofluorescence, anti-Rv1579, anti-Rv2645 pAbs developed with AF488-anti-IgG and Rhodamine B (RB). RB is a kind of *M.tb* specific fluorescence dye, used as a positive control. Samples with a score less than the cutoff value (0.0580) were designated as “-”, (≥0.0580 and <0.5010) as “+”, (≥0.5010 and <0.8780) as “++”, >0.8780 as “+++”. The numbers (0.5010 and 0.8780) were determined by calculating the 50% and 75% percentile of the Score.

*M.tb* infection mouse model was further used to verify ManLAM aptamer-based IHC. C57BL/6 mice were infected with *M.tb* H37Rv as described in *Materials and Methods*. We generated three polyclonal antibodies (pAbs) which reacted with a unique surface antigen ManLAM from *M.tb* H37Rv, the *M.tb*-specific antigens (Rv2645) (Luo et al., 2015) and (Rv1579) (Qu et al., 2020), respectively. We also applied the commercial polyclonal antibody against *M.tb*-specific Ag85B antigen. Then, IHC utilizing these polyclonal antibodies and ManLAM aptamer were performed to detect the antigens in the lung sections from virulent *M.tb* H37Rv strain infected mouse model. Comparing the lung tissues from mice 28 days post-infection with those of uninfected control mice, the positive signals were observed as brown granular staining for aptamer based IHC (**Figure 4A**, 2^nd^ lane) or polyclonal antibodies in the lung tissue sections from the infected mouse (**Figure 4A**, Lanes 3–5), but not in the uninfected lung sections (**Figure 4B**, Left Panels). These results were consistent with AFS staining results for the same lung tissue sections (**Figures 4A, B**, 1^st^ Lane). The positive signal produced by ManLAM aptamer-based IHC (score = 0.368) was similar to those of ManLAM polyclonal antibody (score = 0.262) or polyclonal antibodies against *M.tb*-Ag85B (score = 0.356) and Rv2645 (score = 0.227) (**Figure 4A**) in the same mouse lung tissues. And similar results between aptamer-based IHC and antibody-based IHC were observed for TB patient samples (ManLAM aptamer-based IHC: score = 0.748; *M.tb*-Ag85B pAb: score = 0.229; Rv2645 pAb: score = 0.172**;** **Supplementary Figure 2**).

Next, we further used immunofluorescence method (*Materials and Methods*) to compare aptamer-based immunofluorescence and antibody-based immunofluorescence for TB diagnosis. The AF488 labeled ManLAM aptamer and AF488 labeled anti rabbit-IgG against rabbit anti-Rv2645 and anti-Rv1579 antibodies were used to detect the *M.tb* antigens in the lung sections from *M.tb* infected mouse. Similarly, we observed the positive signals produced by AF488 labeled aptamer or by anti-Rv2645 and anti-Rv1579 pAbs, respectively as shown in **Figure 4C**. According to the results, both aptamer–based IHC and aptamer-based immunofluorescence were confirmed to be able to detect *M.tb* antigens in mouse infection model with high effectiveness, feasibility and easy production.

## Discussion

A pathological examination plays an important role in the confirmation of a diagnosis of tuberculosis, especially for smear- and culture-negative TB cases. Actually, there have been many studies evaluating the importance of *M.tb* antigens by antibodies based immunocytochemistry, such as Ag85B, MPT64, mycobacterial HSP65, PstS1 (38 kDa antigen), and ESAT-6 (Ince et al., 2011; Goel et al., 2012; Feng et al., 2014; Che et al., 2016). As is known to all, these antigens exhibit in different patterns during the infection of TB. For instance, in the necrotic area of granulomas, Ag85B expression is readily detectable whereas MPT64 and PstS1 expression is mainly found in granuloma cells and ESAT-6 expression is found in white cells from cerebrospinal fluid (Ince et al., 2011; Ihama et al., 2012; Feng et al., 2014; Che et al., 2016). It was previously reported that IHC performed on paraffin-embedded tissues using antibody directed against Ag85B showed higher sensitivity than AFS staining (50.5%, 53/105 vs. 31.4%, 33/105; χ² = 7.877, *P* = 0.005) (Che et al., 2016). The combined sensitivity of antibody-based IHC and AFS staining was 59.0% with the improved accuracy of the pathological diagnosis of pulmonary tuberculosis (Che et al., 2016).

ManLAM is constantly released from metabolically active or degrading bacterial cells but not dead cells (Maeda et al., 2003), thus exists in both bacteria and its surrounding space. Thus, ManLAM aptamer based IHC (with a sensitivity of 86.38%) showed more effective performance than antibodies-based IHC (for Ag85B antibody-based IHC, with a sensitivity of 50.5%) for detecting *M.tb* antigens and demonstrated good values in the pathological diagnosis of tuberculosis. ManLAM, besides unique of pathogenic mycobacteria, shows subtle difference in their structures between different mycobacteria subspecies. Our ManLAM aptamer was raised specifically for the more virulent *M.tb* Beijing strain, thus could detect more clinically relevant *M.tb* infection. In addition, the constant released ManLAM from live mycobacteria contributes to its wide distribution, so ManLAM aptamer-based IHC detection has shown stronger and higher evident positive signals.

Usually EPTB constitutes about 15% to 20% of all cases of tuberculosis. The confirmation of EPTB has always been a challenge to laboratory personnel. Our present results showed similar AUC value and sensitivity between EPTB and PTB vs non-TB (**Figure 1D**). Furthermore, the IHC could detect PTB, EPTB, and LTBI patients when AFS results were negative for the same samples as demonstrated in **Figures 4A–C**, suggesting that ManLAM-based IHC is more sensitive than the AFS method. The ManLAM aptamer-based IHC has high sensitivity (86.38%) and specificity (92.86%) for diagnosis of PTB and EPTB. The sensitivity of ManLAM-based IHC (86.38%) has increased ~50% compared to that of the conventional AFS (43.01%), which is the only one available laboratory method for antigen detection for TB diagnosis. Our data suggest that aptamer-based IHC TB diagnosis could be used for TB diagnosis including PTB, EPTB, and LTBI, and has high sensitivity and specificity for PTB and EPTB diagnosis. In addition, we observed that the samples for which ManLAM IHC method had nine false negative results are mostly from EPTB patients (8/9) (**Supplementary Table 5**). And as shown in **Figure 1D**, AUC value was 0.7798 for LTBI *vs* non-TB, 0.9159 for EPTB *vs* non-TB, while 0.9167 for PTB *vs* non-TB. So we propose that ManLAM IHC method might be more effective for PTB diagnosis, slightly less effective for EPTB diagnosis and less effective for LTBI diagnosis. More samples are needed for validation by multicenter studies in the future.

In this study, our method revealed much higher sensitivity than *M.tb* culture and AFS, which are explainable by the facts that AFS or culture requires relatively high bacterial load to present positivity, and that poor sample quality and inappropriate operation procedures often leads to false negative results. The sensitivities of *M.tb* culture and AFS were profoundly elevated after combination with ManLAM aptamer-based IHC. Further, our aptamer-IHC diagnostic performance was fair and comparable to those of IGRA and GeneXpert MTB/RIF, which are currently widely applied in TB diagnosis with high sensitivity. However, IGRA and GeneXpert MTB/RIF assays are impeded by the high cost and apparatus requirement, especially in lower-middle income countries (Vadwai et al., 2011; Tortoli et al., 2012; Luabeya et al., 2019). In addition, IGRA appeared to perform poorly in children or in patients with HIV infection (Hormi et al., 2018). In contrast, ManLAM aptamer based IHC directly measures TB antigens in lesion tissues and does not rely on the host immune status and thus holds an advantage for TB diagnosis in immunodeficient or infant patients. With the calculation of IHC score and the optimal cutoff value, which accounted for the background, we believe that background signal could not interfere with the discrimination between TB patients and non-TB patients. ManLAM aptamer-based IHC has advantages of easy sample preparation and low cost compared to antibodies-based IHC, IGRA and GeneXpert MTB/RIF (Vadwai et al., 2011; Tortoli et al., 2012), and is a convenient, feasible and economical diagnostic method for TB.

Moreover, we have evaluated and compared the performance of the ManLAM aptamer-based IHC method with other routine techniques for TB diagnosis, including *M.tb* culture, AFS, IGRA and GeneXpert MTB/RIF. We demonstrated that the sensitivities of these TB diagnosis approaches were dramatically improved from 9.66%~84.38% to 88.64%~97.92% when combined with the IHC assay developed in this study for TB diagnosis. At last but not least, the IHC method has low hardware requirements and can be observed at a lower magnification (10× or 20×) rather than using oil immersion under microscopy. Up to now, we have not found any other better aptamers than ssDNA T9 aptamer for TB diagnosis according to our previous research and literature on the PubMed, Web of Science, and other literature databases. So, we propose that the ssDNA T9 aptamer might be the most suitable aptamer for TB diagnosis application, although the modification of aptamer or combination of multiple aptamers for higher sensitivity by multicenter studies would be needed for further validation in future study.

In sum, we demonstrated the great application value of aptamer based-IHC in the histological diagnosis of TB, using a specific high affinity single-strand DNA aptamer “antibody” T9 directed against ManLAM. As our knowledge, this is the first report about aptamer-based IHC for disease diagnosis. ManLAM-aptamer based IHC not only can be used for TB differential diagnosis, it can also profoundly improve the diagnostic sensitivity when in cooperation with common conventional methods for TB diagnosis, and it will also be helpful for surgical pathology diagnosis.

Looking forward, we anticipate optimization and standardization method for future further study, including larger blinded samples validation, and combination with point of cars testing (POCT), will be performed to make it better applied in clinical diagnosis in the future.

## Data Availability Statement

The original contributions presented in the study are included in the article/**Supplementary Material**. Further inquiries can be directed to the corresponding authors.

## Ethics Statement

The studies involving human participants were reviewed and approved by Ethics Committee of Wuhan University School of Medicine and Ethics Committee of Jin Yin-Tan Hospital. Written informed consent to participate in this study was provided by the participants’ legal guardian/next of kin. The animal study was reviewed and approved by Institutional Animal Care and Use Committee (IACUC) of Wuhan University.

## Author Contributions

YZ and XH carried out experiments and drafted the paper. YZ, XH, and YX performed and analyzed experimental results. YK and JR assisted in the experiments. X-LZ designed experiments and supervised the study. RC, LW, and CH assisted in the specimen procurement and performed the histopathological analysis. All authors contributed to the article and approved the submitted version.

## Funding

This work was supported by the National Grand Program on Key Infectious Disease (2017ZX10201301-006 to X-LZ), the National Key Research and Development Program (2018YFA0507603 to X-LZ), the National Natural Science Foundation of China (22077097, 91740120 to X-LZ), the Medical Science Advancement Program (Basical Medical Sciences) of Wuhan University (TFJC 2018002 to X-LZ), the Natural Science Foundation Project and Technological Innovation Major Project of Hubei Province (2016CFA062, 2016ACA150 to X-LZ), and the Hubei Province’s Outstanding Medical Academic Leader Program (523-276003 to X-LZ).

## Conflict of Interest

The authors declare that the research was conducted in the absence of any commercial or financial relationships that could be construed as a potential conflict of interest.

## Supplementary Material

Supplementary Figure 1 The flow diagram of the study. Details of patients are provided in the appendix. non-TB: other non-TB diseases (e.g., bronchiectasis, lung cancer); TB: Tuberculosis; AFS-/+: acid fast staining negative/positive; Culture-/+: culture negative/negative; IGRA -/+: IGRA positive/negative.

Supplementary Figure 2 Comparison of aptamer- and antibodies-based IHC for TB diagnosis with patient tissue lesions. Images of lesion sections of LNTB patient **(A)** and NTM patient **(B)** detected by aptamer-based IHC (upper panel), IHC with anti-Ag85B (middle panel), and IHC with anti-Rv2645 (lower panel).
