# Supplementary figures and images for: Aptamer Detection of Mycobaterium tuberculosis Mannose-Capped Lipoarabinomannan in Lesion Tissues for Tuberculosis Diagnosis
Source: Front Cell Infect Microbiol. 2021 Mar 15;11:634915. doi: 10.3389/fcimb.2021.634915 (PMC8006938; doi:10.3389/fcimb.2021.634915)

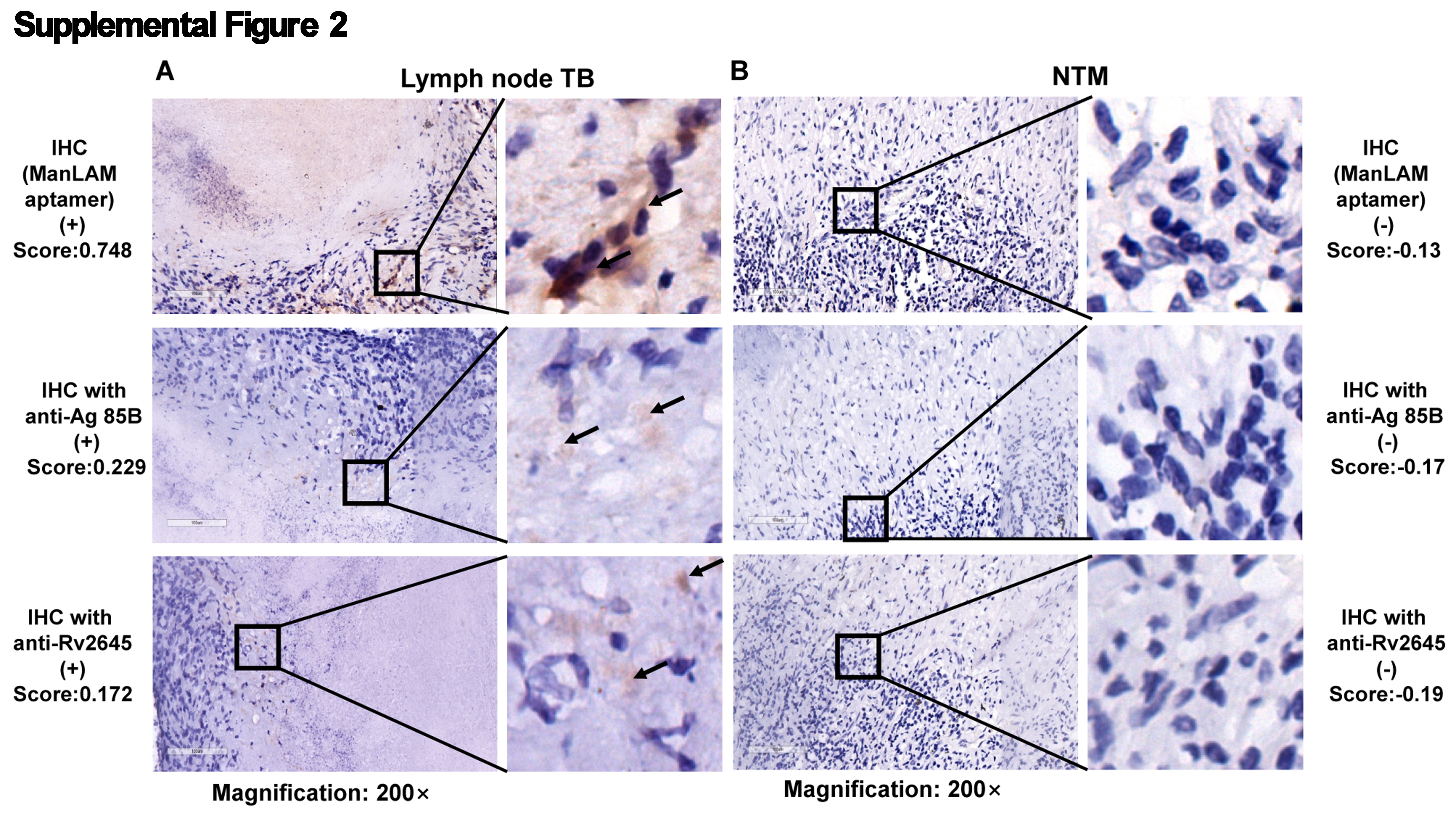

Supplement: Supplementary Figure 1 — The flow diagram of the study. Details of patients are provided in the appendix. non-TB: other non-TB diseases (e.g., bronchiectasis, lung cancer); TB: Tuberculosis; AFS-/+: acid fast staining negative/positive; Culture-/+: culture negative/negative; IGRA -/+: IGRA positive/negative. [file Image_1.tif]

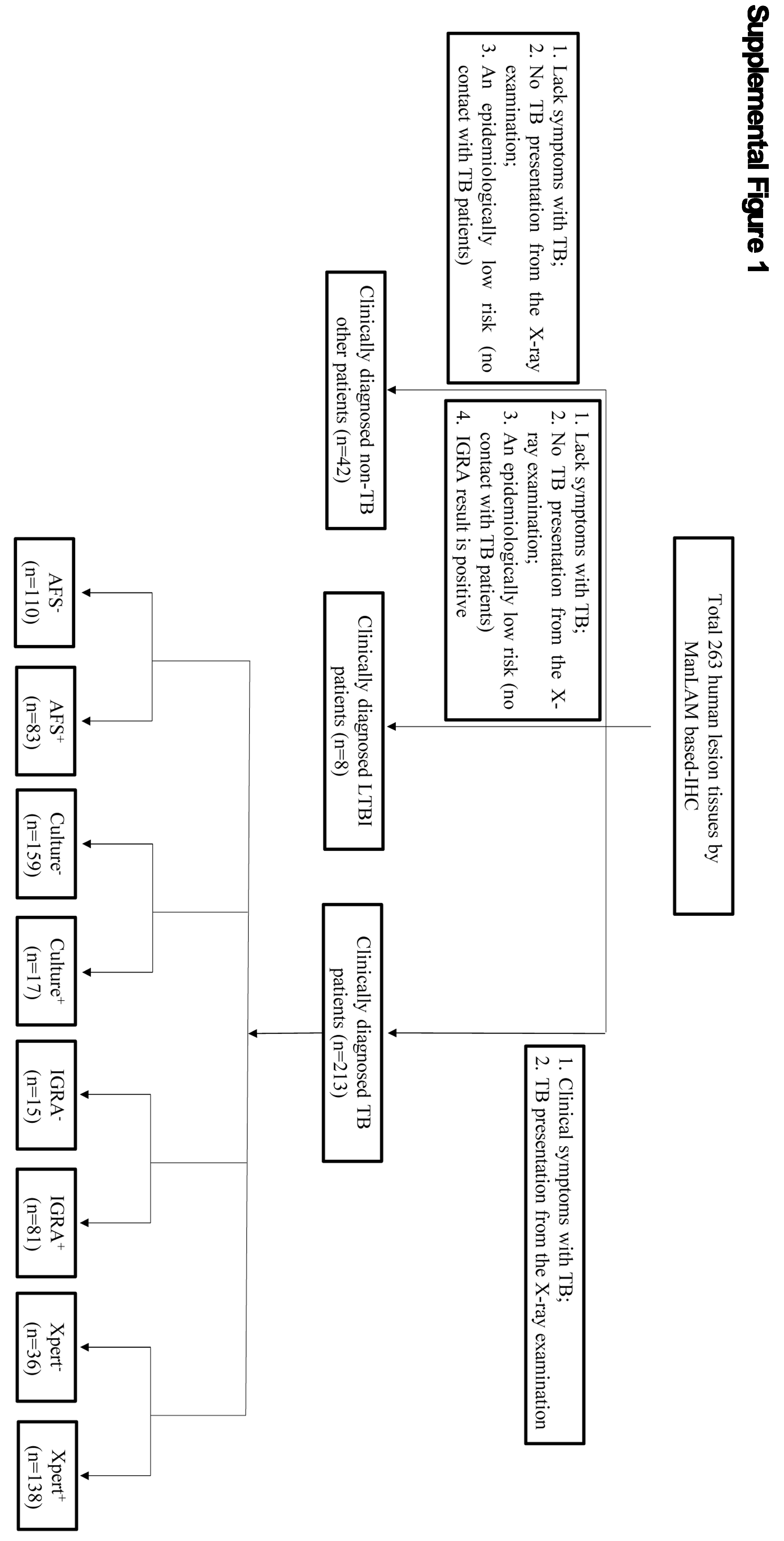

Supplement: Supplementary Figure 2 — Comparison of aptamer- and antibodies-based IHC for TB diagnosis with patient tissue lesions. Images of lesion sections of LNTB patient (A) and NTM patient (B) detected by aptamer-based IHC (upper panel), IHC with anti-Ag85B (middle panel), and IHC with anti-Rv2645 (lower panel). [file Image_2.tif]
