# Supplementary material for: Aptamer Detection of Mycobaterium tuberculosis Mannose-Capped Lipoarabinomannan in Lesion Tissues for Tuberculosis Diagnosis
Source: Front Cell Infect Microbiol. 2021 Mar 15;11:634915. doi: 10.3389/fcimb.2021.634915 (PMC8006938; doi:10.3389/fcimb.2021.634915)
Supplement: Supplementary file 3 [file Table_1.docx]

**Supplementary Table 1. Clinical characteristics of** **study participants.**

| **Assay** | **Aptamer-based IHC for TB diagnosis (tissue samples)** | |  | **Aptamer-based IHC for TB diagnosis (tissue samples)** | | | |  | **Aptamer-based IHC and IGRA for TB diagnosis** |  | **Aptamer-based IHC and GeneXpert for TB diagnosis** |
| --- | --- | --- | --- | --- | --- | --- | --- | --- | --- | --- | --- |
| **Characteristic** | **TB**  **(n =213)** | **Non-TB (n=42)** |  | **PTB**  **(n=38)** | **EPTB**  **(n=175)** | **LTBI**  **(n=8)** | **Non-TB**  **(n=42)** |  | **PTB (n = 14)**  **EPTB (n = 82)** |  | **PTB (n = 30)**  **EPTB (n = 144)** |
| **Age, median (range)** | 31.0  (0.2-86.0) | 48.5  (1.0-72.0) |  | 37.5  (12.0-70.0) | 30.0  (0.2-86.0) | 59.5  (44.0-71.0) | 48.5  (1.0-72. 0) |  | 32.5  (0.2-82.0) |  | 31.0  (0.2-86.0) |
| **Female gender** | 92/213  (43.19%) | 12/42 (28.57%) |  | 17/38  (44.74%) | 75/175  (42.86%) | 4/8  (50.00%) | 12/42  (28.57%) |  | 44/96  (45.83%) |  | 87/174  (50.00%) |
| **Clinical presentations** |  |  |  |  |  |  |  |  |  |  |  |
| TB symptoms | 213/213  (100.00%) | 0/42 |  | 38/38  (100.00%) | 175/175  (100.00%) | 0/8 | 0/42 |  | 96/96  (100.00%) |  | 174/174  (100.00%) |
| Comordities ^a^ | 13/213  (6.10%) | 7/42 (16.67%) |  | 4/38  (10.52%) | 9/175  (5.14%) | 0/8 | 7/42  (16.67%) |  | 6/96  (6.25%) |  | 10/174  (5.75%) |
| Positive HIV infection ^b^ | 3/213  (1.41%) | 2/42  (4.76%) |  | 0/38 | 3/175  (1.71%) | 3/8  (37.50%) | 2/42  (4.76%) |  | 1/96  (1.04%) |  | 3/174  (1.72%) |
| Abnormal Chest X-ray findings | 213/213  (100.00%) | 0/42 |  | 38/38  (100.00%) | 175/175  (100.00%) | 0/8 | 0/42 |  | 96/96  (100.00%) |  | 174/174  (100.00%) |
| **Laboratory findings** |  |  |  |  |  |  |  |  |  |  |  |
| AFS | 83/193  (43.01%) | 0/16 |  | 7/37  (18.92%) | 76/156  (48.72%) | 0/8 | 0/16 |  | 36/85  (42.35%) |  | 80/171  (46.78%) |

**Supplementary Table 1. Clinical characteristics of** **study participants (cont.).**

| Culture | 17/176  (9.66%) | 0/14 |  | 2/32  (6.25%) | 15/144  (10.42%) | 0/8 | 0/14 |  | 11/79  (13.92%) |  | 17/163  (10.43%) |
| --- | --- | --- | --- | --- | --- | --- | --- | --- | --- | --- | --- |
| Positive IGRA ^c^ | 81/96  (84.38%) | 0/11 |  | 13/14  (92.86%) | 68/82  (82.93%) | 8/8  (100.00%) | 0/11 |  | 81/96  (84.38%) |  | 69/80  (86.25%) |
| GeneXpert | 138/174  (79.31%) | 0/9 |  | 13/29  (44.83%) | 125/145  (86.21%) | 0/8 | 0/9 |  | 58/80  (72.50%) |  | 138/174  (79.31%) |

Data are presented as No. of positive/No. of tested (%), median (range).

^a^ Comordities indicates one of the following diseases including diabetes mellitus, malnutrition, cancer, treatment with immunosuppressive medications, end-stage renal disease, anemia, etc., which could lead to compromised immune status.

^b^ HIV: human immunodeficiency virus;

^c^ IGRA: Interferon-γ Release Assay

**Supplementary Table 2. Statistical evaluation of the detection rates for different diagnosis methods.**

| **Study Population** | **Sensitivity** | | ***P* value**  **(A *vs* B)** |
| --- | --- | --- | --- |
|  | **A: Routine TB diagnosis Methods** | **B: Combination of ManLAM aptamer-based IHC**  **with other routine TB diagnosis methods** |  |
| **Total TB** | Culture: 9.66% (17/176) | [IHC + Culture]: 88.64% (156/176) | <0.0001 |
|  | AFS: 43.01% (83/193) | [IHC + AFS]: 91.19% (176/193) | <0.0001 |
|  | IGRA: 84.38% (81/96) | [IHC + IGRA]: 97.92% (94/96) | 0.0023 |
|  | GeneXpert: 79.31% (138/174) | [IHC + GeneXpert]: 95.98 (167/174) | <0.0001 |
|  | [Culture + AFS]: 44.10% (86/195) | [IHC + Culture + AFS]: 90.77% (177/195) | <0.0001 |
|  | [Culture+ AFS + IGRA+GeneXpert]:83.90% (172/205) | [IHC+Culture+AFS+ IGRA + GeneXpert]:97.07% (199/205) | <0.0001 |

TB: tuberculosis; IGRA: interferon gamma release assay; AFS: acid fast staining; IHC: ManLAM based-IHC. The differences in sensitivity were statistically evaluated with proportion test (R prop.test). Sensitivity = (No. of Test positive TB patients)/ (No. of TB patients).

**Supplementary Table 3. Clinical profiles for thirteen patients with a positive result of ManLAM-based IHC and a negative result of IGRA.**

| **No.** | **Gender** | **Age (years)** | **Diagnosis** | **Laboratory findings** | | | | | **TB Symptoms** | **Chest X-ray** | **Comordities ^c^** |
| --- | --- | --- | --- | --- | --- | --- | --- | --- | --- | --- | --- |
|  |  |  |  | AFS | TB  Culture | GeneXpert | Histopathology | HIV |  |  |  |
| 1 | Male | 0.2 | EPTB | NA | NA | NA | + | - | + | + | - |
| 2 | Male | 0.2 | EPTB | + | - | + | + | - | + | + | + |
| 3 | Female | 0.3 | EPTB | + | NA | + | - | - | + | + | + |
| 4 | Female | 0.3 | EPTB | - | - | - | - | - | + | + | + |
| 5 | Male | 0.6 | EPTB | + | - | + | + | - | + | + | - |
| 6 | Female | 0.6 | EPTB | NA | NA | NA | + | - | + | + | - |
| 7 | Female | 0.7 | EPTB | + | - | + | + | - | + | + | - |
| 8 | Male | 1 | EPTB | - | NA | NA | + | - | + | + | - |
| 9 | Female | 13 | EPTB | - | - | - | - | - | + | + | - |
| 10 | Female | 27 | EPTB | - | - | - | - | - | + | + | - |
| 11 | Male | 27 | EPTB ^a^ | + | - | + | + | - | + | + | + |
| 12 | Male | 40 | EPTB | - | - | - | - | - | + | + | - |
| 13 | Male | 60 | PTB ^b^ | - | - | - | + | - | + | + | - |

“-”: negative; “+”: positive; NA: not available

^a^ EPTB: extrapulmonary tuberculosis;

^b^ PTB: pulmonary tuberculosis;

^c^ Comordities indicates one of the following diseases including diabetes mellitus, malnutrition, cancer, treatment with immunosuppressive medications, end-stage renal disease, anemia, etc., which could lead to compromised immune status.

**Supplementary Table 4. Clinical profiles for twenty-nine patients with positive results of ManLAM-based IHC and negative results of GeneXpert.**

| **No.** | **Gender** | **Age (years)** | **Diagnosis** | **Laboratory findings** | | | | | **TB Symptoms** | **Chest X-ray** | **Comordities ^c^** |
| --- | --- | --- | --- | --- | --- | --- | --- | --- | --- | --- | --- |
|  |  |  |  | AFS | TB  Culture | IGRA | Histopathology | HIV |  |  |  |
| 1 | Female | 0.3 | EPTB ^a^ | - | - | - | - | - | + | + | + |
| 2 | Male | 1 | EPTB | + | + | + | + | - | + | + | - |
| 3 | Female | 13 | EPTB | - | - | - | + | - | + | + | - |
| 4 | Female | 22 | PTB ^b^ | - | - | NA | + | - | + | + | + |
| 5 | Male | 22 | EPTB | - | - | - | + | - | + | + | - |
| 6 | Male | 24 | PTB | - | - | NA | + | - | + | + | - |
| 7 | Male | 25 | EPTB | + | - | + | - | - | + | + | - |
| 8 | Female | 26 | PTB | + | NA | NA | + | - | + | + | - |
| 9 | Female | 26 | PTB | - | - | NA | + | - | + | + | - |
| 10 | Female | 27 | EPTB | - | - | - | - | - | + | + | - |
| 11 | Female | 28 | PTB | - | - | + | - | - | + | + | - |
| 12 | Male | 29 | EPTB | - | NA | NA | + | - | + | + | - |

**Supplementary Table 4. Clinical profiles for twenty-nine patients with positive results of ManLAM-based IHC and negative results of GeneXpert (cont.).**

| 13 | Male | 30 | PTB | - | - | NA | + | - | + | + | - |
| --- | --- | --- | --- | --- | --- | --- | --- | --- | --- | --- | --- |
| 14 | Female | 31 | EPTB | - | - | + | - | - | + | + | - |
| 15 | Male | 32 | PTB | - | - | + | + | - | + | + | - |
| 16 | Male | 37 | PTB | - | - | + | + | - | + | + | - |
| 17 | Female | 38 | EPTB | - | NA | + | + | - | + | + | - |
| 18 | Male | 38 | PTB | - | - | + | + | - | + | + | - |
| 19 | Female | 40 | EPTB | + | - | NA | + | - | + | + | - |
| 20 | Male | 40 | EPTB | - | - | - | - | - | + | + | - |
| 21 | Female | 41 | PTB | - | - | + | + | - | + | + | - |
| 22 | Female | 49 | EPTB | - | - | + | - | - | + | + | - |
| 23 | Male | 50 | EPTB | - | - | + | - | + | + | + | + |
| 24 | Male | 52 | EPTB | - | - | + | - | - | + | + | - |
| 25 | Female | 56 | PTB | + | - | NA | - | - | + | + | - |
| 26 | Male | 59 | PTB | + | - | NA | - | - | + | + | - |
| 27 | Male | 59 | EPTB | - | - | NA | + | - | + | + | - |

**Supplementary Table 4. Clinical profiles for twenty-nine patients with positive results of ManLAM-based IHC and negative results of GeneXpert (cont.).**

| 28 | Male | 60 | PTB | - | - | - | + | - | + | + | - |
| --- | --- | --- | --- | --- | --- | --- | --- | --- | --- | --- | --- |
| 29 | Male | 70 | PTB | - | - | NA | + | - | + | + | - |

“-”: negative; “+”: positive; NA: not available

^a^ EPTB: extrapulmonary tuberculosis;

^b^ PTB: pulmonary tuberculosis;

^c^ Comordities indicates one of the following diseases including diabetes mellitus, malnutrition, cancer, treatment with immunosuppressive medications, end-stage renal disease, anemia, etc., which could lead to compromised immune status.

**Supplementary Table 5. Clinical profiles for nine patients with negative results of ManLAM-based IHC and positive results of IGRA.**

| **No.** | **Gender** | **Age (years)** | **Diagnosis** | **Laboratory findings** | | | | | **TB Symptoms** | **Chest X-ray** | **Comordities ^c^** |
| --- | --- | --- | --- | --- | --- | --- | --- | --- | --- | --- | --- |
|  |  |  |  | AFS | TB  Culture | GeneXpert | Histopathology | HIV |  |  |  |
| 1 | Male | 19 | EPTB | + | + | + | + | - | + | + | - |
| 2 | Female | 21 | EPTB ^a^ | + | + | + | + | - | + | + | - |
| 3 | Male | 23 | EPTB | NA | - | + | + | - | + | + | - |
| 4 | Male | 25 | EPTB | - | - | - | + | - | + | + | - |
| 5 | Male | 30 | EPTB | - | - | - | + | - | + | + | - |
| 6 | Female | 44 | EPTB | - | - | - | - | - | + | + | - |
| 7 | Male | 63 | EPTB | + | + | + | + | - | + | + | - |
| 8 | Female | 64 | PTB ^b^ | - | - | - | - | - | + | + | - |
| 9 | Male | 70 | EPTB | - | - | + | + | - | + | + | - |

“-”: negative; “+”: positive; NA: not available

^a^ EPTB: extrapulmonary tuberculosis;

^b^ PTB: pulmonary tuberculosis;

^c^ Comordities indicates one of the following diseases including diabetes mellitus, malnutrition, cancer, treatment with immunosuppressive medications, end-stage renal disease, anemia, etc., which could lead to compromised immune status.

**Supplementary Table 6. Clinical profiles for seventeen patients with negative results of ManLAM-based IHC and positive results of GeneXpert.**

| **No.** | **Gender** | **Age (years)** | **Diagnosis** | **Laboratory findings** | | | | | **TB Symptoms** | **Chest X-ray** | **Comordities ^c^** |
| --- | --- | --- | --- | --- | --- | --- | --- | --- | --- | --- | --- |
|  |  |  |  | AFS | TB  Culture | IGRA | Histopathology | HIV |  |  |  |
| 1 | Male | 0.8 | EPTB | + | - | NA | + | - | + | + | - |
| 2 | Male | 16 | EPTB ^a^ | - | - | NA | + | - | + | + | - |
| 3 | Male | 19 | EPTB | + | + | + | + | - | + | + | - |
| 4 | Female | 21 | EPTB | + | + | + | + | - | + | + | - |
| 5 | Male | 22 | PTB ^b^ | + | - | NA | + | - | + | + | - |
| 6 | Male | 23 | EPTB | NA | - | + | + | - | + | + | - |
| 7 | Male | 30 | EPTB | + | + | NA | + | - | + | + | - |
| 8 | Male | 30 | EPTB | - | - | + | + | + | + | + | - |
| 9 | Female | 31 | PTB | - | NA | NA | + | - | + | + | - |
| 10 | Female | 37 | EPTB | - | NA | NA | + | - | + | + | - |
| 11 | Male | 39 | PTB | + | - | NA | + | - | + | + | - |
| 12 | Male | 48 | EPTB | - | - | NA | + | - | + | + | - |

**Supplementary Table 6. Clinical profiles for seventeen patients with negative results of ManLAM-based IHC and positive results of GeneXpert (cont.).**

| 13 | Male | 50 | PTB | - | - | NA | - | - | + | + | - |
| --- | --- | --- | --- | --- | --- | --- | --- | --- | --- | --- | --- |
| 14 | Male | 62 | EPTB | - | - | NA | + | - | + | + | - |
| 15 | Male | 63 | EPTB | + | + | + | + | - | + | + | - |
| 16 | Male | 67 | EPTB | - | - | NA | + | - | + | + | - |
| 17 | Male | 70 | EPTB | - | - | + | + | - | + | + | - |

“-”: negative; “+”: positive; NA: not available

^a^ EPTB: extrapulmonary tuberculosis;

^b^ PTB: pulmonary tuberculosis;

^c^ Comordities indicates one of the following diseases including diabetes mellitus, malnutrition, cancer, treatment with immunosuppressive medications, end-stage renal disease, anemia, etc., which could lead to compromised immune status.
